# Targeted Transforaminal Epidural Blood Patch for Postdural Puncture Headache in Patients with Postlaminectomy Syndrome

**DOI:** 10.1155/2019/1968314

**Published:** 2019-06-19

**Authors:** Yu Na Choi, Sang Ji Kang, Jin Deok Joo, Yu Mi Kim, Jang Hyeok In, Yoo Jung Park

**Affiliations:** ^1^Department of Anesthesiology and Pain Medicine, Bucheon ST. Mary's Hospital, College of Medicine, The Catholic University of Korea, Bucheon 14647, Republic of Korea; ^2^Department of Anesthesiology and Pain Medicine, Saint Vincent's Hospital, College of Medicine, The Catholic University of Korea, Suwon 16247, Republic of Korea

## Abstract

Postdural puncture headache is a leak of cerebrospinal fluid that lowers intracranial pressure and usually presents as a positional headache. If conservative treatments are not successful, the epidural blood patch is the gold standard of the treatment for dural puncture. The interlaminar approach is the most commonly used technique for an epidural blood patch. This case report describes a patient who was treated with a transforaminal epidural blood patch for postdural puncture headache following an acupuncture procedure on his lower back after two epidural blood patches using an interlaminar approach had failed. The patient underwent an acupuncture therapy for management of chronic low back pain due to postlaminectomy syndrome. After the procedure, the patient had a severe headache and the conservative treatment was not effective. The two interlaminar epidural blood patches at the L2–3 level and at the L3–4 level were failed. We performed transforaminal epidural blood patch at the L3–4 and L4–5 levels on the left side, the site of leakage in the MRI myelogram. His symptoms finally subsided without complication. This case demonstrates that targeted transforaminal epidural blood patch is a therapeutic option for the treatment of postdural puncture headache when epidural blood patch using an interlaminar approach is ineffective.

## 1. Introduction

Postdural puncture headache (PDPH) is caused by reduced cerebrospinal fluid (CSF) pressure due to the loss of CSF from the epidural space through the dural puncture [1]. Typically, the postural headache worsens with sitting or standing and improves with lying down [2]. The headache can be accompanied by neck stiffness, tinnitus, and nausea and vomiting [3]. Risk factors include female sex, young age, pregnancy, repeated puncture, and low body mass index. Needle size, design, and the technique used also affect the risk [2].

Acupuncture, which is used to treat acute or chronic pain, can also cause PDPH. The depth of insertion of the acupuncture needle varies from a few millimeters to several centimeters, and the insertion direction varies from perpendicular to angled. Therefore, the tip of the needle can be placed in muscles or overlie other structures, such as nerves and the pleura [4].

Conservative therapies such as bed rest, hydration, caffeine, and acetaminophen are commonly used to treat PDPH. If conservative care is ineffective, an epidural blood patch (EBP) is often used. Traditionally, the interlaminar approach is the most common technique used for an EBP.

Here, we describe a patient who was treated with a transforaminal EBP for PDPH following an acupuncture procedure on his lower back after two EBPs using an interlaminar approach had failed.

## 2. Case

A 27-year-old man was referred to our pain clinic with an 8-day history of postural headache. He had undergone L4–5 laminectomy 7 years earlier to treat a herniated nucleus pulposus at the L4–5 level. He underwent acupuncture therapy 9 days before presenting to our clinic to manage chronic lower back pain caused by postlaminectomy syndrome. The acupuncture treatment involved the insertion of a needle 10 cm long from the center and laterally. After the procedure, the patient had a severe headache, which had a numeric rating scale of 7 to 9 out of 10. He felt pain upon sitting, together with fullness of the ears and neck stiffness. On assuming a supine position, his symptoms resolved within 5 min. The physical and neurological examinations were normal. He was diagnosed with PDPH and placed on bed rest. In the clinic, an EBP was performed via an interlaminar approach at the L2–3 level. However, his symptoms did not improve. Consequently, he was referred to our hospital.

In our clinic, under fluoroscopic guidance, we performed an interlaminar EBP using an 18-guage Tuohy needle (Tae-Chang Industrial, Seoul, Republic of Korea) at the L2–3 level. After successful loss of resistance, 15 mL sterile autologous blood was injected at the L2–3 level in the midline without a catheter. However, this failed to relieve the patient's symptoms, which still occurred within 5 min of standing or sitting up. The patient continued conservative care, including bed rest, hydration, and taking acetaminophen. Brain magnetic resonance imaging (MRI) looking for the site of CSF leakage was unremarkable, while a MRI myelogram showed an abnormal fluid signal intensity in the left lumbar area, along the left paraspinal muscle and soft tissues at the L3–4–5 level, probably due to CSF leakage. Because of the persistent symptoms, a repeat fluoroscopically guided interlaminar EBP was performed 4 days after the initial procedure using 15 mL autologous blood at the L4–5 level. The day after this procedure, the patient reported slight symptom relief but again experienced the same severe headache, posterior neck tenderness, and fullness of the ears after standing for 15 min. The conservative care was continued.

**Figure 1:**
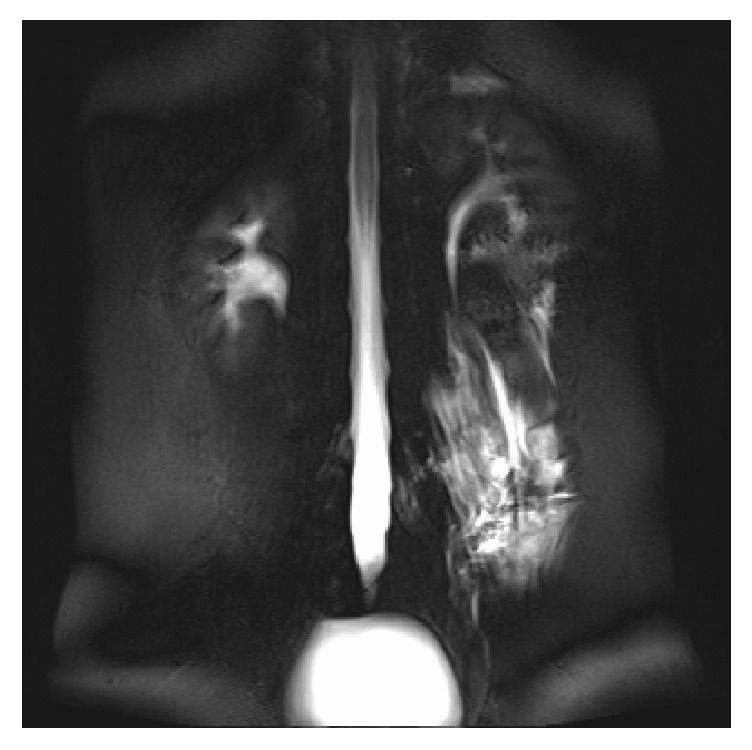
The MRI myelogram shows an abnormal fluid signal intensity in the left lumbar area, along the left paraspinal muscle and soft tissues at the L3–4–5 level, probably due to CSF leakage.

**Figure 2:**
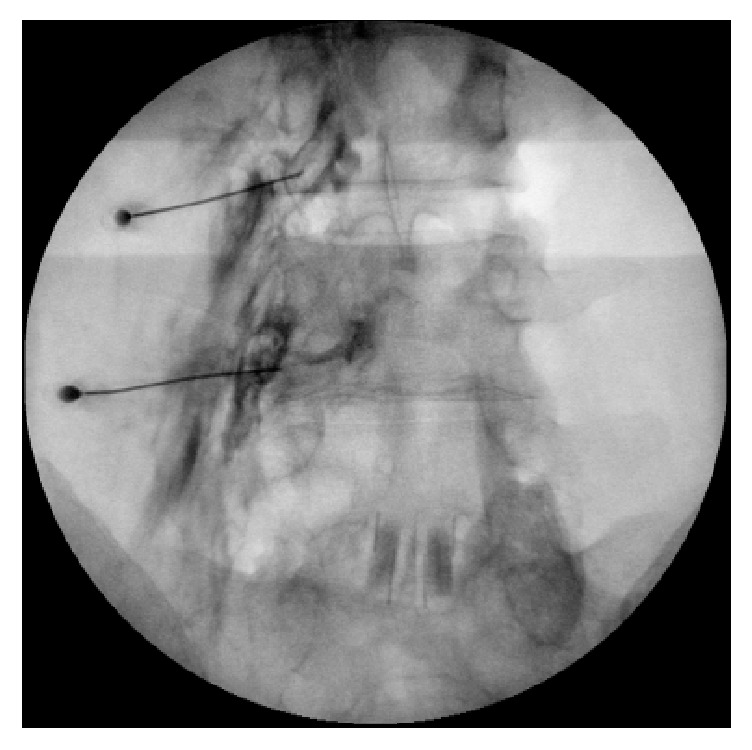
Fluoroscopic image of the epidural contrast injected through the left L3–4 and L4–5 foramens.

The patient's postural headaches persisted for 4 days, so we decided to perform a transforaminal EBP at the L3–4 and 4–5 levels on the left side, the site of leakage in the MRI myelogram (Figure 1). Under fluoroscopic guidance, a 22-gauge needle (Hakko, Japan) was inserted with a transforaminal approach and then contrast was injected at the left L3–4 and L4–5 levels (Figure 2). Then, after epidural spread was seen, 3 mL sterile autologous blood was injected into the transforaminal epidural space at each level. During injection, the patient's vital signs were monitored and the injection was stopped when the patient began to feel pressure in his lower back, but no pain or paresthesias were reported.

After the transforaminal EBP, his headache and other symptoms finally subsided without complications. The patient was symptom-free the next morning. After 4 days, the patient was discharged. At the 2-week follow-up, he reported being headache-free and his activities had returned to normal.

## 3. Discussion

August Bier first described spinal headache in 1898. One study found 10 cases in 453 patients who had 7,963 spinal injections. Although the exact mechanism of PDPH remains unknown, it might be caused by reduced CSF pressure due to the loss of CSF in the epidural space through the dural puncture site [1]. One possible cause of PDPH is that decreased CSF pressure leads to traction and tension across the pain-sensitive dural sinuses in the upright position [1, 5]. Therefore, the treatment is to seal the hole, which is how EBP works [6]. A second possible cause is vasodilation of the intracranial vessels. When the CSF pressure drops suddenly, the intracranial vessels vasodilate to maintain a constant intracranial volume [7]. Therefore, the use of a vasoconstrictor such as caffeine or theophylline is effective for treating PDPH. Both mechanisms likely have a role in the genesis of PDPH.

When PDPH occurs, several conservative treatments are commonly used, such as hydration, bed rest, and administration of acetaminophen or caffeine. For patients who do not respond to conservative treatment, EBP is the gold standard [5, 8]. The EBP is performed by injecting the patient's autologous blood into the epidural space around the site of suspected CSF leakage.

There are two theories about the mechanism by which EBP resolves PDPH. The “plug” theory for symptom resolution proposes that the injected autologous blood stops the loss of CSF by forming a clot over the defect in the meninges. In the absence of continued CSF loss, regeneration of CSF restores the CSF pressure and alleviates the PDPH [9]. The “pressure patch” theory posits that the injected blood or other fluid (crystalloid or colloid) elevates the subarachnoid CSF pressure by compressing the dura, which increases the pressure in the epidural space. According to this theory, PDPH is alleviated by reducing traction on pain-sensitive structures and decreasing vascular dilation. The relief of headache is not related to the hole in the dura, but persistent relief requires the elevated pressure to be maintained [9].

There is no consensus as to whether targeted or blind EBP is best. Although some studies have suggested that targeted EBP is more effective, targeted EBP in the cervical and thoracic regions has an increased risk for complications. Therefore, it is reasonable to try a blind approach first, followed by targeted EBP if the symptoms persist and the site of CSF leakage is identified [10].

Of the two approaches to the epidural space, an interlaminar approach is almost always used for the EBP [8, 11]. There are only a few reported cases using the transforaminal approach for EBP. In one report, transforaminal EBP was performed in a patient with a chronic headache secondary to spontaneous intracranial hypotension because it allowed the placement of blood at the exact site of the CSF leak when an interlaminar approach was impractical because of a previous laminectomy [12]. In another report, an 11-year-old boy underwent a transforaminal EBP to manage a refractory PDPH that was secondary to a ventral epidural CSF leak determined using radiological imaging. The PDPH resolved with the transforaminal EBP after three failures with an interlaminar EBP [13].

In our patient, fluoroscopically guided, blind interlaminar EBP at the L2–3 level failed. We hypothesized that the blind interlaminar EBP at the L2–3 level was likely ineffective because it failed to reach the site of the CSF leak. The targeted interlaminar EBP at the L4–5 level was also ineffective, possibly because the epidural blood did not spread due to adhesions caused by postoperative epidural fibrosis after the laminectomy. We believe that the targeted transforaminal EBP allowed us to inject blood directly at the site of the CSF leak seen on the MRI myelogram, while avoiding the epidural adhesions. We recommend the targeted transforaminal technique when the conventional interlaminar approach has failed or when a ventral or far-lateral CSF leak is identified with imaging studies.

## 4. Conclusion

The targeted interlaminar EBP in a patient who had laminectomy can be ineffective, possibly because the epidural blood did not spread due to adhesions caused by postoperative epidural fibrosis. This case demonstrates that targeted transforaminal EBP can be a therapeutic option for the treatment of PDPH in the patient with postlaminectomy syndrome when EBP using an interlaminar approach is ineffective. We suggest the targeted transforaminal technique when the conventional interlaminar approach has failed or when a ventral or far-lateral CSF leak is identified with imaging studies.

## Data Availability

The data used to support the findings of this study are included within the article.
